# Neuroprotective, Anti-Amyloidogenic and Neurotrophic Effects of Apigenin in an Alzheimer’s Disease Mouse Model

**DOI:** 10.3390/molecules18089949

**Published:** 2013-08-19

**Authors:** Le Zhao, Jun-Li Wang, Rui Liu, Xiao-Xu Li, Jian-Fei Li, Lu Zhang

**Affiliations:** 1 College of Life and Environmental Sciences, Minzu University of China, 27 South Street, Zhongguancun, Beijing 100081, China; E-Mails: zhaole@bjciq.gov.cn (L.Z.); rna829@sina.com (X.-X.L); lzg006688@163.com (J.-F.L); zhanglu.goodluck@163.com (L.Z); 2 Institute of Materia Medica, Chinese Academy of Medical Sciences & Peking Union Medical College, Beijing 100050, China

**Keywords:** Alzheimer’s disease, amyloid-β peptide, apigenin, oxidative stress, neurotrophin

## Abstract

Alzheimer’s disease (AD) is a neurodegenerative disorder characterized by extracellular senile plaques and intracellular neurofibrillary tangles in the brain. Amyloid-β peptides (Aβ) are considered to play a critical role in the onset and progression of AD. Apigenin (4',5,7-trihydroxyflavone) is a pharmacologically active agent. Even though some evidence suggests that it has potential neuroprotective effects, no preexisting study has reported any therapeutic effects of apigenin in AD models. In the present study, we examined the effects of apigenin on cognitive function in APP/PS1 double transgenic AD mice and explored its mechanism(s) of action. Three-month oral treatment with apigenin rescued learning deficits and relieved memory retention in APP/PS1 mice. Apigenin also showed effects affecting APP processing and preventing Aβ burden due to the down-regulation of BACE1 and β-CTF levels, the relief of Aβ deposition, and the decrease of insoluble Aβ levels. Moreover, apigenin exhibited superoxide anion scavenging effects and improved antioxidative enzyme activity of superoxide dismutase and glutathione peroxidase. In addition, apigenin restored neurotrophic ERK/CREB/BDNF pathway in the cerebral cortex. In conclusion, apigenin may ameliorate AD-associated learning and memory impairment through relieving Aβ burden, suppressing amyloidogenic process, inhibiting oxidative stress, and restoring ERK/CREB/BDNF pathway. Therefore, apigenin appears to represent an alternative medication for the prevention and/or therapy of AD.

## 1. Introduction

Alzheimer’s disease (AD) is one of the most prevalent neurodegenerative diseases worldwide. Its pathological features include synaptic degeneration, senile plaques and neurofibrillary tangles, which ultimately lead to neuronal dysfunction and loss [1]. The major components of senile plaque are amyloid-β peptides (Aβ), which are derived from proteolytic cleavage of amyloid precursor protein (APP). β-site APP-cleaving enzyme 1 (BACE1) is mainly responsible for β-cleavage of APP *in vivo* [2]. By mediating β-secretase cleavage of APP, it generates the soluble APP N-terminal fragment, sAPPβ, and a membrane-tethered C-terminal fragment of 99 amino acids (β-CTF), which undergoes further processing by γ-secretase to release the APP intracellular domain and Aβ fragments.

Accumulation of Aβ, which may result from its overproduction or defective clearance, is considered to play a pivotal role in the onset and progression of AD [3,4]. Insofar as it can be ascertained, Aβ peptides are directly neurotoxic by mechanisms involving oxidative stress, mitochondrial dysfunction, apoptosis, and hyperphosphorylation of tau, thereby causing neurofibrillary tangle formation and abnormal neuronal functions [1,5,6,7]. Aβ also disturbs normal synaptic function and cognitive capability [8,9], accompanied by loss of neurotrophins (NTs), including nerve growth factor (NGF), brain-derived neurotrophic factor (BDNF), neurotrophin 3 (NT3) and NT4/5, as well as the suppression of learning and memory molecular transduction, including the mitogen-activated protein kinases (MAPK) and cAMP response element-binding protein (CREB) [10,11,12].

Apigenin (4',5,7-trihydroxyflavone), a less toxic and non-mutagenic flavone, is widely distributed in many fruits, vegetables such as cabbage, celery and bell pepper, and medicinal herbs including *Elsholtzia rugulosa* [13] and *Carduus crispus* [14]. It has been used for its anxiolytic, sedative and antidepressant effects [15]. Apigenin isolated from *Carduus crispus* showed protection against kainate-induced excitotoxicity by quenching reactive oxygen species (ROS) as well as inhibiting glutathione peroxidase (GSH) depletion in hippocampal neurons [14]. Apigenin isolated from *Elsholtzia rugulosa* exhibited protective effects against Aβ-induced toxicity by regulating redox imbalance and strengthening barrier function in rat cerebral microvascular endothelial cells [16]. Recently, apigenin has been reported to alleviate learning and memory deficits and express the neurovascular protection by decreasing oxidative damage, improving cholinergic neuronal transmission, and preserving the blood-brain barrier integrity in Aβ_25–35_ intracerebroventricularly injected mice [17]. The inhibition of the influx of extracellular Ca^2+^ and release of intracellular Ca^2+^ in the rat thoracic aorta [18], and the antagonism of NMDA and γ-aminobutyric acid (GABA) receptor channels [19] in neurons might be part of the protective mechanisms. Further, we demonstrated that apigenin exerted neuroprotection against Aβ-mediated toxicity mainly through the mechanisms of regulating redox imbalance, preserving mitochondrial function, inhibiting p38 MAPK-MAPKAP kinase-2 (MK2)-heat shock protein 27 (HSP27) and stress-activated protein kinase (SAPK)/c-Jun N-terminal kinase (JNK)-c-Jun pathways, and depressing apoptosis [20].

Despite the investigation for apigenin of its protective effects by interacting with Aβ peptides [16,17,20,21], knowledge of therapeutic effects on Aβ-related cognitive impairment and potential mechanisms is limited. Thus, as a part of our ongoing evaluation program to evaluate the protective potential of natural compounds, we have investigated the effect of apigenin on cognitive function in a double-transgenic mouse model of AD. Moreover, we investigated the mechanisms underlying the efficacy of the compound.

## 2. Results and Discussion

As an extension of previous research, the current study further clarified the beneficial effects of apigenin on AD-associated pathology. Our findings indicate a clear rescue of learning and memory deficits in apigenin-treated APP/presenilin-1 (PS1) double-transgenic mice. Apigenin also showed effects on affecting APP processing and preventing Aβ burden involving the decrease of BACE1 and β-CTF levels, the relief of Aβ deposition, and the reduction of insoluble Aβ levels. Moreover, apigenin exhibited superoxide anion scavenging effects and improved antioxidative enzyme activity of superoxide dismutase (SOD) and GSH-Px. In addition, apigenin protected neurotrophic extracellular signal-regulated kinases (ERK)/CREB/BDNF pathway in cerebral cortex. These observations are correlated with a prospective neuroprotective, anti-amyloidogenic and neurotrophic effects in AD deficits.

### 2.1. Apigenin Treatment Prevents Learning and Memory Deficits in APP/PS1 Mice

**Figure 1:**
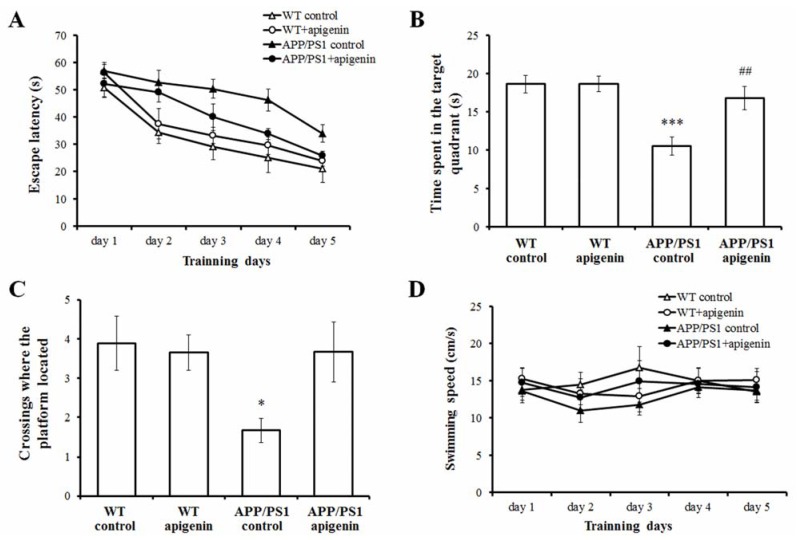
Long-term apigenin treatment improves learning and memory deficits in APP/PS1 mice. (**A**) Apigenin treatment attenuates the learning deficits compared with vehicle-treated APP/PS1 mice. (**B**) Apigenin increases the time a mouse spent in the target quadrant in the probe test. *** *p* < 0.001 *vs.* WT control mice, ^##^ *p* < 0.01 *vs.* APP/PS1 control mice. (**C**) Apigenin affects the numbers of crossings where the platform had been located in the probe test. * *p* < 0.05 *vs.* WT control mice. (**D**) No significant differences are observed in motor function of the apigenin-treated mice and the vehicle controls as reflected by the swimming speed. All data are presented as mean ± S.E.M., n = 9 mice per group.

MWM test is one of the most widely accepted behavioral tests monitoring the spatial learning and memory capabilities. Spatial learning was assessed by the time required to find the hidden platform (escape latency). The dose of apigenin was selected based on the preliminary studies. Figure 1A shows the results of all mice during acquisition training. Repeated-measures ANOVA revealed a significant day effect on escape latency (*F*_(4,128)_ = 32.111, *p* < 0.001) within the groups, indicating that mice in different groups showed different spatial learning capability during the 5-day acquisition training. There is also a significant treatment effect (*F*_(3,32)_ = 10.524; *p* < 0.001) on the escape latency, and subsequent comparisons further suggested that 40 mg/kg apigenin treatment is effective in rescuing spatial learning deficits in APP/PS1 mice (*p* < 0.05).

Probe trials were conducted to assess the memory retention in the last training session. APP/PS1 control mice spent less time searching for the platform and showed less numbers of crossings in the target quadrant (where the platform had been located) compared to the WT control ones (*p* < 0.001, *p* < 0.05, Figure 1B and C). Apigenin-treated APP/PS1 mice spent more time searching in the target quadrant relative to APP/PS1 vehicle control (*p* < 0.01, Figure 1B). The numbers of crossings where the platform located of apigenin-treated APP/PS1 mice are more than those of the untreated APP/PS1 mice, but the values did not reach significance. These results demonstrated that apigenin improved the spatial memory capability in the APP/PS1 mouse model.

Toxic effects of Aβ on the cholinergic system and cognitive function are variable and due to the differences in the sites of administration and experimental models [22,23,24,25]. In order to investigate the prospective therapeutic value of apigenin on cognitive impairment in the correlation with neurodegeneration of AD, we applied transgenic mice that overexpress a chimeric mouse/human APP695 harboring the Swedish mutation K595N/M596L and a mutant human PS1 carrying the exon 9-deleted variant under the control of independent mouse prion protein promoter elements, directing transgene expression predominantly to central neurons [26]. These APP/PS1 double transgenic mice develop cognitive impairment with increasing age [27]. In the present study, after apigenin treatment, the APP/PS1 mice were tested behaviorally at the age of 7 months.

Here, apigenin, by oral gavage of 40 mg/kg/day, improved spatial learning effectively across the 5- day acquisition training period. In the subsequent memory probe trial, the APP/PS1 mice receiving apigenin treatment performed much better in searching the target quadrant and the site where the platform was located as compared to the APP/PS1 vehicle controls. To exclude the possibility that the improvement of apigenin on spatial learning and memory in APP/PS1 mice was not due to sensorimotor abnormality, we analyzed their swimming ability. The result showed that there was no significant difference in swimming speed among the groups across the five training days (Figure 1D). In addition, 40 mg/kg apigenin treatment in WT mice did not show any significant effect on learning and memory capabilities, which indicates that long-term oral administration of apigenin had no effect on performance in normal mice, but can rescue learning deficits and relieve memory retention in APP/PS1 mice.

As far as existing research is concerned, apigenin is a biochemically active flavonoid with known treatment on cognition parameters in AD-related models. Apigenin showed learning and memory improvement properties for short-term treatment with respect to the neurovascular unit protection in Aβ_25–35_-induced amnesic mouse model [17]. Moreover, apigenin-7-glucoside, an apigenin derivative, also slowed the cognitive impairment with chronic treatment as in AD by inhibiting the overexpression of COX-2 and iNOS in age- and LPS-induced amnesia [28]. Therefore, considering the cognitive improvement properties of apigenin presented in this and previous studies, it is quite possible that apigenin might contribute to learning and memory therapeutic innovations in AD.

### 2.2. Apigenin Treatment Relieves Aβ Burden in APP/PS1 Mice

**Figure 2:**
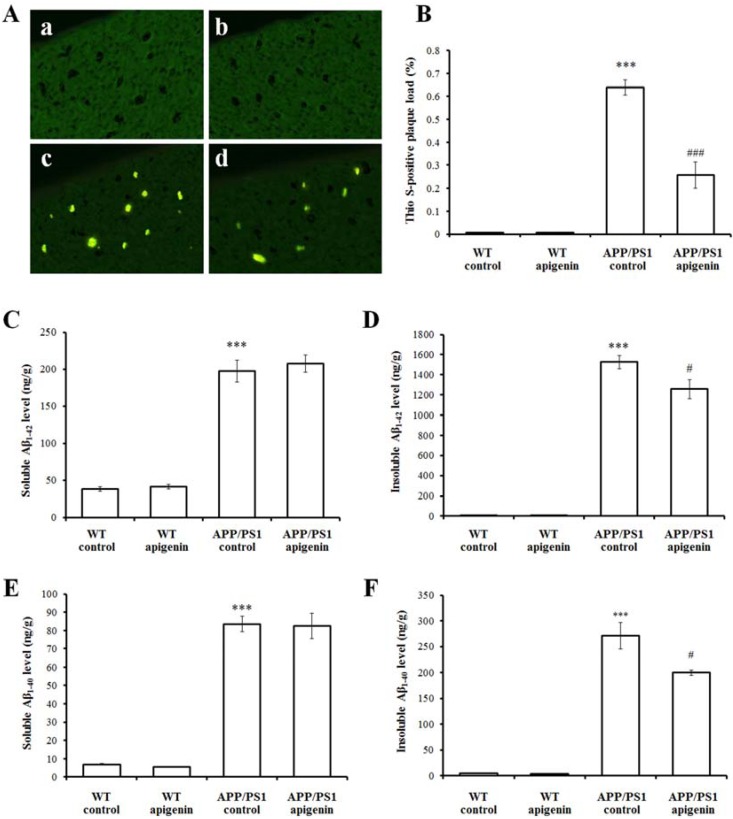
Apigenin treatment relieves Aβ burden in APP/PS1 mice. (**A**) Representative images of Thio S positive compact plaques staining in cortex of wild-type mice and APP/PS1 mice (400 ×). **a**, WT vehicle control; **b**, apigenin-treated WT; **c**, APP/PS1 vehicle control; **d**, apigenin-treated APP/PS1. (**B**) Quantitative analysis of Thio S positive compact plaques staining. *** *p* < 0.001 *vs.* vehicle-treated WT mice, *^###^ p* < 0.001 *vs.* APP/PS1 control mice. Data are presented as mean ± S.E.M., n = 4. (**C**–**F**) ELISA analysis of soluble or insoluble Aβ_1–42_ (**C**,**D**), _1–40_ (**E**,**F**) levels in extracts of cerebral homogenates of WT and APP/PS1 mice. *** *p* < 0.001 *vs.* WT control mice, ^#^ *p* < 0.05 *vs.* APP/PS1 control mice. All data are presented as mean ± S.E.M., n = 4.

One of the key features of AD pathology is the extracellular accumulation of Aβ in neuritic plaques. In order to evaluate whether the improvement in mouse behavior was correlated with the change of this neuropathological marker, we analyzed the burden of Aβ deposits and the changes of Aβ levels in the brain of the APP/PS1 mice. Fibrillar amyloid deposits were visualized by Thioflavin S (Thio S) staining. No detectable Aβ deposits stained with Thio S in the cortex was found in vehicle and apigenin-treated WT mice [Figure 2A(a,b)]. In contrast, a large number of Thio S-positive plaques were observed in APP/PS1 mice [*p* < 0.001, Figure 2A(c) and B]. Compared with the APP/PS1 control mice, slices of apigenin-treated APP/PS1 mice showed a reduction in the Thio S-positive area (*p* < 0.001, Figure 2A and B), indicating that apigenin reduced fibrillar amyloid deposits. To further assess the effect of apigenin on Aβ production, we performed an analysis of cerebral Aβ levels by ELISA. Quantitative analysis revealed that there were significant differences in the soluble or insoluble Aβ levels among the WT mice and APP/PS1 mice (*p* < 0.001, Figure 2C–F). Apigenin treatment lowered insoluble Aβ_1–40_ and Aβ_1–42_ levels (*p* < 0.05, Figure 2D and F) in APP/PS1 mice.

Due to a characteristic neuropathological hallmark of AD, treatment strategies aimed at reducing the generation and accumulation of Aβ should therefore be theoretically possible. APP/PS1 mice start to develop Aβ plaque deposition as early as two months of age [25], and exhibit moderate levels of Aβ deposition at the age of five months [29]. In the present study, we confirmed the effect on Aβ burden of apigenin in APP/PS1 double transgenic mice. After three months of treatment, evaluation of the pathological brains of apigenin treated APP/PS1 mice showed a remarkable reduction in Aβ plaque burden in the cortex. Further, the Aβ lowering properties of apigenin by measuring soluble and insoluble Aβ species in apigenin-treated and -untreated APP/PS1 mice are characterized. Data showed that the chronic treatment with apigenin in APP/PS1 mice reduced cerebral levels of insoluble Aβ_1–40_ and Aβ_1–42_ by 20% and 19.8%, respectively, while apigenin showed a non-significant effect for reducing soluble Aβ levels. In addition, apigenin has been shown to reduce Aβ toxicity in neuronal AD cells without sufficient effect on lowering Aβ_1–42_ secretion [20]. Because the Aβ pellets in the insoluble fractions include aggregated and fibrillar substances, and Thio S staining mainly detects β-pleated sheet and fibrillar amyloid plaques, we believe that apigenin reduces fibrillar and compacted Aβ plaques preferentially over soluble Aβ that are reported to show less significant correlations in amyloidogenic processing of APP, accumulation of Aβ aggregation, and aberrant increase of BACE-1 activity than insoluble Aβ in AD-related disease [30].

The possibility that apigenin may have an effect on the N-terminal cleavage of Aβ by BACE is being further evaluated.

### 2.3. Apigenin Treatment Reduces BACE1 Level and β-Amyloidogenesis in APP/PS1 Mice

**Figure 3:**
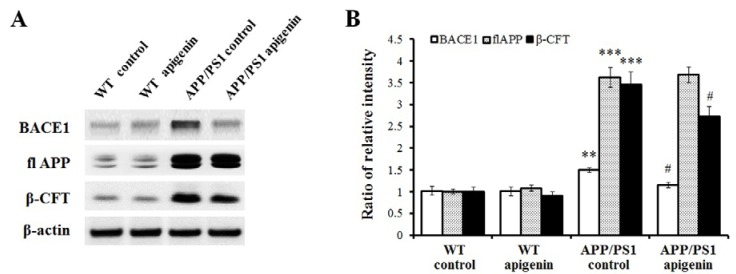
Apigenin treatment inhibits β-amyloidogenic process in APP/PS1 mice. (**A**) Representative immunoblots for cerebral BACE1, flAPP and β-CFT in APP/PS1 mice. (**B**) Quantitative analysis for BACE1, flAPP and β-CFT. Data are presented as mean ± S.E.M., n = 4. ** *p* < 0.01, *** *p* < 0.001 *vs*. WT control mice, ^#^ *p* < 0.05 *vs.* APP/PS1 control mice.

To further investigate the possibility that apigenin treatment could improve learning and memory capabilities and relieve Aβ burden through affecting APP processing in APP/PS1 mice, we conducted immunoblot analysis to assess the levels of BACE1, full-length APP (flAPP), and its β-cleaved fragments, β-CTF. Results showed that levels of BACE1 and β-CTF in cerebral cortex of apigenin-treated APP/PS1 mice were significantly lowered than those of APP/PS1 controls (*p* < 0.05, *p* < 0.05, Figure 3). Meanwhile, transgene-derived overexpression of human flAPP was not affected by chronic administration of apigenin in APP/PS1 mice. Apigenin treatment did not reduce BACE1 and β-CTF levels in WT control mice, indicating that apigenin may not suppress baseline levels of BACE1 and its following β-amyloidogenesis in normal brains, but under AD conditions.

Consistent with the above findings of Aβ deposition and Aβ levels, we have examined the effects of apigenin on amyloid-related pathology in AD mice. Following chronic apigenin treatment, we found in the cortex of APP/PS1 mice that: (i) Aβ levels and deposition were significantly decreased, and (ii) BACE1 protein level as well as its β-cleaved fragments C99 level was significantly down-regulated. Our findings of potential β-amyloidogenesic effects of apigenin are consistent with previous observations of its apoptotic attenuation on cell death induced by insulin fibrils though anti-amyloidogenic activities [31]. Therefore, apigenin might have the capability to affect APP processing and decrease Aβ levels in AD brains.

It is reported that BACE1 is a stress-response protein [32], and thus its protein level might be modulated [33]. In this study, we observed BACE1 level reduction treated with apigenin. In addition to apigenin-induced BACE1 protein level decrease in the AD mouse model, we also found that BACE1 related amyloidogenic process is inhibited in AD brain. These findings are consistent with the effects of apigenin on the inhibition of BACE-1 enzymatic activity in a concentration dependent manner using cell-free and cell-based assay systems [34]. The mechanism involved in BACE1 down-regulation by apigenin remains to be confirmed. It has been suggested that genetic inactivation of tumor necrosis factor receptor-mediated NF-κB signal transduction results in the decrease of BACE1 levels, ultimately leading to Aβ reduction [35]. Combined with previous studies that evidenced NF-κB inactivation by apigenin treatment [36,37], we postulate that apigenin possesses the capability to suppress NF-κB transduction, which is also a pivotal pathway responsible for BACE1 inhibition. Taken collectively, affecting APP processing and decreasing Aβ burdens treated with apigenin may be partly explained for ameliorating memory impairments associated with AD.

### 2.4. Apigenin Treatment Decreases Oxidative Stress in APP/PS1 Mice

The high metabolic rate and reduced capability for cellular regeneration make the brain susceptible to oxidative stress. Oxidative damage is often postulated to be an preceeding event in AD pathogenesis [38,39]. In addition, oxidative stress is also well-recognized as a pivotal mechanism of Aβ toxicity in which over-production of ROS alters Aβ peptide kinetics leading to an increased Aβ burden [6,40,41].

**Figure 4:**
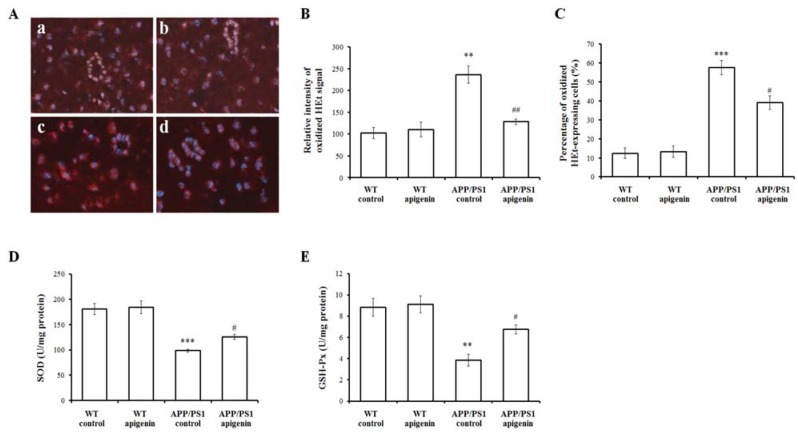
Apigenin treatment inhibits oxidative stress in APP/PS1 mice. (**A**) Representative images of oxidized HEt staining in cortex of WT mice and APP/PS1 mice (200 ×). **a**, WT vehicle control; **b**, apigenin-treated WT; **c**, APP/PS1 vehicle control; **d**, apigenin-treated APP/PS1. (**B**,**C**) Quantitative analysis of the relative cytosolic oxidized HEt intensity and the percentage of oxidized ethidium-expressing cells. Data are presented as mean ± S.E.M., n = 3. ** *p* < 0.01, *** *p* < 0.001 *vs.* WT control, ^#^ *p* < 0.05, ^##^ *p* < 0.01 *vs.* APP/PS1 vehicle control. (**D**,**E**) The activities of oxidative stress markers, SOD (**D**) and GSH-Px (**E**), in extracts of cerebral homogenates in APP/PS1 mice. Data are presented as mean ± S.E.M., n = 4. ** *p* < 0.01, *** *p* < 0.001 *vs.* WT vehicle control mice, ^#^ *p* < 0.05 *vs.* APP/PS1 vehicle control mice.

Oxidized hydroethidine (HEt) represents the superoxide anion level of the cerebral cortex. Under double exposure of oxidized HEt and Hoechst nuclear staining, oxidized HEt was localized in a relatively weak signal in cell cytosol of the cortical slices of WT and apigenin control mice (Figure 4Aa,b), but oxidized HEt signals in the cytosol were widely and intensively distributed in the APP/PS1 mouse slice (Figure 4Ac), and the relative cytosolic oxidized HEt intensity and the percentage of oxidized HEt-positive cells were increased by 2.31- and 4.62 fold, respectively (*p* < 0.01, *p* < 0.001, Figure 4B and C). In the slices of apigenin-treated APP/PS1 mice, there was a significant decrease of oxidized HEt signals (Figure 4Ad), and the relative cytosolic oxidized HEt intensity and the percentage of oxidized HEt-positive cells were decreased by 45.84% and 31.95% (*p* < 0.01, *p* < 0.05, Figure 4B and C), respectively.

Changes of oxidative markers, such as GSH and SOD, can be detected prior to brain Aβ deposition both in humans [42] and AD mice [39,43]. In the present study, we found a decrease of GSH and SOD activity in 7-month-old APP/PS1 mice compared with WT control mice (*p* < 0.05, *p* < 0.05, Figure 4D and E). We detected changes in these two markers in APP/PS1 mice with apigenin treatment, suggesting that sufficient effect of apigenin might be involved in oxidative amelioration in APP/PS1 mouse model.

Apigenin is a bioactive compound and possesses the actions of scavenging ROS. It has been reported that the presence of C2-C3 double bond on the C ring is critical for this biological activity of flavonoid compounds [44]. In particular, the ROS clearance activity seems to depend partly on the number of hydroxyl groups. The free radical scavenging activity of apigenin against hydrogen peroxide and ROS has been demonstrated in many studies [17,45,46]. What’s more, our previous study has evidenced that apigenin processes antioxidant effect through scavenging ROS in a transgenic AD cell model [16]. Here, besides that apigenin scavenged superoxide anions, it increased the antioxidative capacity in the brain. Combined with these findings, we speculate that apigenin provides sufficient antioxidant effect in neuroprotection in these AD models.

### 2.5. Apigenin Treatment Restores ERK/CREB/BDNF Pathway of Cerebral Cortex in APP/PS1 Mice

**Figure 5:**
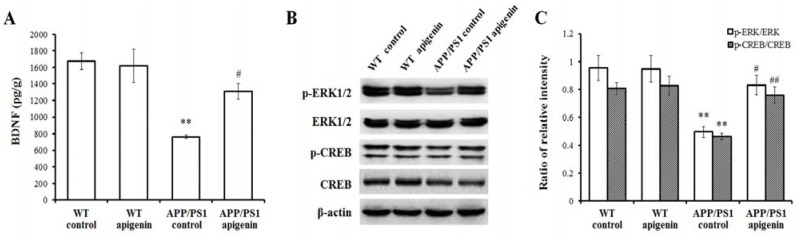
Apigenin treatment protects ERK/CREB/BDNF pathway of cerebral cortex in APP/PS1 mice. (**A**) Cerebral BDNF level in APP/PS1 mice detected by ELISA. (**B**,**C**) Representative immunoblots for p-ERK, total ERK, p-CREB and CREB and quantitative analysis for them. Data are presented as mean ± S.E.M., n = 4. ** *p* < 0.01 *vs.* WT control mice, ^#^ *p* < 0.05, ^##^ *p* < 0.01 *vs.* APP/PS1 control mice.

Neurotrophins, especially BDNF, are critical molecules that have been implicated in the pathophysiology of AD. Several studies have reported that neurotrophic BDNF deficiency begins in the early stages of AD and eventually causes the depression of learning and memory signal transduction, such as the MAPK/ERK and the following transduction to CREB [10,47]. As shown in Figure 5, our findings showed that cortical BDNF level, phosphorylated ERK1/2 and phosphorylated CREB expressions of cerebral cortex were suppressed in APP/PS1 mice (*p* < 0.01). After long-term apigenin treatment, coupled with the elevation of BDNF level, enhanced phosphorylated ERK1/2 and CREB expression were detected in the cerebral cortex (*p* < 0.05–0.01).

Learning and memory are controlled at the molecular level in the brain [48]. There are several major signal pathways participating in the process, one of which is MAPK. In the MAPK family, ERK1/2 converges to signal to CREB [49], the transcription factor, and then CREB binds to the promoter regions of many genes associated with memory and synaptic plasticity [50]. BDNF is one of the many effectors of CREB phosphorylation regulation and participates in the learning and memory processes [51,52,53], and ERK/CREB/BDNF can be seriously suppressed by sublethal Aβ treatment in cortical neuronal loss [54]. In the present study, phosphorylation of ERK1/2 and CREB proteins were shown to be lowered in 7-month old APP/PS1 mouse brain. Apigenin treatment rescued the depression of ERK1/2/CREB/BDNF pathway, which is in good agreement with a previous study indicating that CREB/BDNF signal is a molecular therapeutic pathway in the treatment of double transgenic AD mice [10]. Thus, accompanied with the cognitive improvement, apigenin also showed the effect on the modulation of synaptic transduction of ERK/CREB/BDNF in the cortical cholinergic system.

## 3. Experimental

### 3.1. Reagents

Apigenin with 98% HPLC purity was purchased from Huike Botanical Development Company (Xi’an, China). Unless otherwise stated, all other reagents were purchased from Sigma Chemical Company (St. Louis, MO, USA).

### 3.2. APP/PS-1 Transgenic Mice and Drug Treatment

APP/PS1 double transgenic mice were purchased from The Jackson Laboratory (Bar Harbor, ME, USA, strain name, B6C3-Tg (AβPPswe, PSEN1dE9) 85Dbo/J; stock number, 004462). Age-matched wild-type (WT) littermates were used as controls. Mice were housed 3–5 per cage with free access to standard food and water and were maintained under standard laboratory conditions. The mice were allowed to acclimatize to the laboratory conditions before testing. The experiment was carried out in compliance with The Guidelines for Animal Care and Use of China, and the experimental protocols were approved by the animal ethics committee of Minzu University of China.

In the present study, four month-old APP/PS1 mice and littermate WT mice were divided into the following treatment groups: WT controls (n = 9, four males and five females), WT + apigenin 40 mg/kg (n = 9, five males and five females), APP/PS1 controls (n = 9, five males and four females), and APP/PS1 + apigenin 40 mg/kg (n = 9, five males and four females). Apigenin was dissolved in distilled water containing 5% sodium carboxyl methyl cellulose (CMC-Na) at a concentration of 10 mg/mL. Apigenin-treated mice received apigenin dissolved in CMC-Na by oral gavage 5 day/week at a dose of 40 mg/kg body weight once a day. Control mice received 5% CMC-Na in the same manner as vehicle controls. Treatment was continued for 12 weeks.

After behavioral testing was completed, the mice were divided into two parallel experiments. (1) Six mice of each group were anaesthetized and blood was collected by cardiac puncture followed by transcardial perfusion with 30–40 mL phosphate buffered saline (PBS). The brains were quickly removed. One hemi-brain was snap frozen in liquid nitrogen. The other was fixed and stored in 4% paraformaldehyde in PBS and later dehydrated. The effects of apigenin on oxidative enzyme activity, neurotrophic molecule level, Aβ burden and APP processing were tested. (2) The remaining mice of each group were used for the detection of the neurovascular oxidative stress. They were administered HEt intravenously through the right jugular vein and then fixed with 4% paraformaldehyde by transcardial perfusion. The division of mice into treatment groups and selection of mice to be sacrificed within each group were both done randomly.

### 3.3. Morris Water Maze Performance

The effect of apigenin treatment on spatial learning and memory was assessed by Morris water maze (MWM) testing at the age of 7 months. A stainless pool with a diameter of 100 cm and a height of 50 cm containing a submerged escape platform (10 cm in diameter) 1.5 cm below the water surface was used. The water temperature was kept at 23 ± 1 °C. The acquisition task consisted of five consecutive days of testing with four trials per day. In each trial, the time required to escape onto the hidden platform was recorded as escape latency. The mice were given a maximum of 60 s to find the hidden platform. If a mouse failed to find the platform within 60 s, the training was terminated, a maximum score of 60 s was assigned, and the mouse was manually guided to the hidden platform. A single probe trial was conducted on the last trial of the fifth day. The platform was removed and the mice were placed into the pool from the quadrant opposite to the training quadrant. Mice were allowed to swim for 60 s. The time the mice spent in the platform quadrant and numbers of crossings where the platform had been located were recorded. Swimming speed was measured by dividing the path length by the time to find the platform. Only mice with a swimming speed over 8 cm/s in all trials were included in the analysis to discard occasional floating mice. The treatments were continued during the water maze task. One hour before the MWM test, mice were treated with vehicle or apigenin.

### 3.4. Thio S Staining for Aβ Deposition Measurement

Slices from frontal cortex were stained for detection of Aβ plaques as previously described [55]. After slices were incubated in a 1% aqueous solution of Thio S for 10 min, they were rinsed in 80% and 95% ethanol, and then distilled water. To quantify Thio S staining, acquisition of images was performed in a single session using a Nikon camera mounted on a Nikon Eclipse 80i microscope (Nikon, Tokyo, Japan). Image analysis was performed using Image-Pro Plus 5.1 (Media Cybernetics, Bethesda, MD, USA). The percent area occupied by Thio S in the cortex was calculated for 3 equidistant sections per mouse.

### 3.5. Detection of Oxyradicals *in Situ*

The changes of oxidative stress in the brain were investigated by in situ detection of oxidized HEt [17,47]. After HEt was intravenous injected, it is selectively oxidized to ethidium by superoxides in the CNS parenchyma. Briefly, mice were anesthetized with 45 mg/kg sodium pentobarbital, and administered intravenously through the right jugular vein 100 μL of HEt (1 mg/mL diluted in PBS; Molecular Probes, Eugene, OR, USA). One hour after HEt injection, mice were perfused with 4% paraformaldehyde by transcardial perfusion. Series of 10 μm thick coronal sections were cut through frontal cortices. The sections were incubated with 2 μM Hoechst 33342 (Dojindo Laboratory, Kumamoto, Japan) in PBS. Photomicrographs of slices were taken using double exposures to oxidized HEt (excitation 510 nm, emission 600 nm) and Hoechst 33342 (excitation 346 nm, emission 460 nm). The percentage of oxidized HEt-expressing cells relative to total cells stained with Hoechst and the percentage increase of fluorescent intensity relative to background values were calculated.

### 3.6. ELISA Analyses for Cerebral Soluble and Insoluble Aβ Levels and BDNF Contents

The levels of soluble and insoluble Aβ in the brain of APP/PS1 mice were quantified according to the procedures as described previously [55]. The cerebral tissue of the hemi-brain were weighed and homogenized in 5 vol/wt of Tris-buffered saline (TBS) containing a cocktail of protease inhibitors. One aliquot of cerebral sample was suspended in 2% sodium dodecyl sulfate (SDS) containing protease inhibitors and centrifuged at 100,000 g for 60 min at 4 °C. The supernatant fraction was collected to be used for the soluble Aβ detection. The remaining sodium dodecyl sulfate-insoluble pellet was sonicated and dissolved in 70% formic acid, and centrifuged at 100,000 g for 60 min. The supernatant was collected to be used for the insoluble Aβ detection. The soluble and insoluble Aβ levels were determined by the commercially human Aβ_1-40/42_ ELISA kits (BioSource, Camarillo, CA, USA) according to the manufacturer’s instructions. Protein concentrations were determined by a BCA kit (Pierce Biotechnology, Rockford, IL, USA). Data obtained in brain homogenates were expressed as ng/mg total protein.

Another aliquot of cerebral sample of hemi-brain was homogenized with ultrasonication and centrifuged at 4 °C for 10 min, 13,000 rpm. The supernatants were recovered and used for detection according to the manufacturer’s directions for BNDF. Protein concentrations were determined by a BCA kit (Pierce Biotechnology). Data are represented as pg/g wet tissue.

### 3.7. Cerebral Oxidative Stress Measurement

The activities of superoxide dismutase (SOD) and glutathione peroxidase (GSH-Px) in the cerebral homogenates were measured using commercial assay kits (Nanjing Jiancheng Company, Nanjing, China) according to the manufacturer's directions.

### 3.8. Western Blot Analysis

Part of the brain tissues was used to determine protein expression in cerebral cortex homogenate. The antibodies used in this experiment were as follows: anti-ERK1/2 (polyclonal antibody, 1:500, Cell Signal), anti-p-ERK1/2 (polyclonal antibody, 1:400, Cell Signal), anti-CREB (polyclonal antibody, 1:400, Cell Signal), anti-p-CREB (polyclonal antibody, 1:400, Cell Signal), anti-BACE1 (polyclonal antibody, 1:500, Cell Signal), anti-APP C-terminal recognizing the C-terminus (amino acids 676–695) of human APP 695) to detect full-length APP/C-terminal fragments (polyclonal antibody, 1:1000, Sigma-Aldrich), and anti-β-actin (monoclonal antibody 1:2000, Cell Signal). Membranes were washed several times with TBST prior to incubation with HRP-conjugated secondary antibody (1:1000, ZSGB-Bio, China) for 45 min at room temperature. After subsequent washes in TBST, the protein bands were visualized using an ECL^TM^ detection kit (GE Healthcare, Piscataway, NJ, USA). Relative optical densities and areas of bands were quantified using an image densitometer. The densitometric plots of the results were normalized to the intensity of the actin band.

### 3.9. Statistical Analysis

All data are represented as the mean ± S.E.M. The *P* values of less than 0.05 were considered statistically significant. Statistical analyses were performed using the SPSS software (Version 16.0; SPSS, Inc., Chicago, IL, USA). Treatment differences in the escape latency in MWM task were analyzed using two-factor analysis of variance with repeated measures on one factor. Tukey’s *post hoc* test was used if the treatment was significant in ANOVA. The other studies were analyzed using one-way ANOVA followed by an appropriate *post-hoc* test to analyze the difference.

## 4. Conclusions

In conclusion, our study demonstrates the therapeutic value of apigenin in APP/PS1 double transgenic mouse model. Although additional investigation is required, the present findings provide preclinical evidence that oral apigenin may ameliorate AD-associated learning and memory impairment through relieving Aβ burden, suppressing amyloidogenic process, inhibiting oxidative stress, and restoring ERK/CREB/BDNF pathway of cerebral cortex in APP/PS1 mice. Therefore, apigenin appears to offer an alternative medication for the prevention and/or therapy of AD.
